# Impact of Incretin-Based Therapies on Adipokines and Adiponectin

**DOI:** 10.1155/2021/3331865

**Published:** 2021-10-07

**Authors:** Habib Yaribeygi, Mina Maleki, Stephen L. Atkin, Tannaz Jamialahmadi, Amirhossein Sahebkar

**Affiliations:** ^1^Research Center of Physiology, Semnan University of Medical Sciences, Semnan, Iran; ^2^Urology and Nephrology Research Center, Shahid Beheshti University of Medical Sciences, Tehran, Iran; ^3^Weill Cornell Medicine Qatar, Doha, Qatar; ^4^Department of Food Science and Technology, Quchan Branch, Islamic Azad University, Quchan, Iran; ^5^Department of Nutrition, Faculty of Medicine, Mashhad University of Medical Sciences, Mashhad, Iran; ^6^Applied Biomedical Research Center, Mashhad University of Medical Sciences, Mashhad, Iran; ^7^Biotechnology Research Center, Pharmaceutical Technology Institute, Mashhad University of Medical Sciences, Mashhad, Iran; ^8^School of Pharmacy, Mashhad University of Medical Sciences, Mashhad, Iran

## Abstract

Adipokines are a family of hormones and cytokines with both pro- and anti-inflammatory effects released into the circulation to exert their hormonal effects. Adipokines are closely involved in most metabolic pathways and play an important modulatory role in lipid and carbohydrate homeostasis as they are involved in the pathophysiology of most metabolic disorders. Incretin-based therapy is a newly introduced class of antidiabetic drugs that restores euglycemia through several cellular processes; however, its effect on adipokines expression/secretion is not fully understood. In this review, we propose that incretin-based therapy may function through adipokine modulation that may result in pharmacologic properties beyond their direct antidiabetic effects, resulting in better management of diabetes and diabetes-related complications.

## 1. Introduction

Diabetes mellitus (DM) is the most prevalent metabolic disorder globally [1]. This chronic metabolic disease results in dysregulation of metabolic pathways towards injurious pathways such as the hexosamine pathway, fatty acid beta-oxidation and oxidative stress, polyol pathways, and glycation end products [2]. This metabolic dysregulation contributes to the development of cardiovascular complications and diabetic microvascular complications [3–5]. In diabetes, the physiological balance of many cellular modulators such as adipokines is disturbed, and this may be an important underlying cause for the further development of diabetes-related complications [6]. Adipokines are a large family of inflammatory cytokines originating from adipocytes and fatty tissues that modulate metabolic pathways [7, 8] associated with obesity, metabolic syndrome, insulin resistance, and DM [9–11]. As a result, normalizing adipokine expression/circulatory levels of these bioactive molecules contributes to normalizing body metabolism and preventing diabetes-related complications [12].

Incretin-based therapy is a newly introduced class of medication that has hypoglycemic effects through several pathways [13–17]. They have multiple pharmacological effects on many intracellular mechanisms and tissues, but their role in modulating adipokines and adipocyte-derived cytokines is unclear. If incretin-based therapy were to function through adipokine modulation, this might result in pharmacologic properties beyond their direct antidiabetic effects that may result in better management of diabetes and diabetes-related complications. Thus, this review has provided an updated analysis on the possible impact of incretin-based antidiabetic drugs on adipocyte-derived peptides.

## 2. Incretin-Based Antidiabetic Drugs

**Figure 1:**
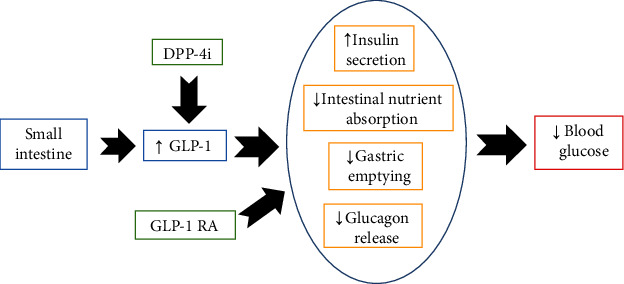
Schematic mechanism of action of incretin-based medications.

Incretins are a group of intestinal metabolic hormones including glucagon-like peptide-1 (GLP-1) and gastric inhibitory peptide (GIP), which affect their hypoglycemic effects through several pathways, including glucagon release inhibition, insulin secretion, delayed gastric emptying, appetite suppression, reducing intestinal nutrients absorption, improving lipid metabolism, and promoting pancreatic *β*-cells' function [13–17] (Figure 1). These peptides act through specific receptors such as the GLP-1 receptor (GLP-1R), which are members of G-protein coupled receptors mainly located on pancreatic *β*-cells [15, 18]. Their activation is followed by increased production of cAMP (cyclic adenosine monophosphate), cellular depolarization, and intracellular calcium augmentation leading to glucose-dependent insulin release from pancreatic *β*-cells [15, 18].

Two main classes of antidiabetic agents have been developed based on incretin hormones; GLP-1 receptor agonists (RA), and dipeptidyl peptidase-4 inhibitors (DPP-4i) (Table 1) [13, 19]. GLP-1ra modulates their hypoglycemic effects by direct binding to the GLP-1R, while DPP-4i inhibits the breakdown of endogenous GLP-1 [13, 19]. GLP-1 is naturally metabolized by a protease called dipeptidyl peptidase-4 (DPP-4) [20, 21]. DPP-4 inhibitors and GLP-1RA both result in hypoglycemic effects, although they show differences in bodyweight reduction and the risk of adverse effects [21, 22] (Table 1).

## 3. Adipokines

Adipose tissue has a prominent role in maintaining metabolic balance in the human body [24]. It is primarily recognized as an energy store [24], but increasingly it has been shown to have biological activities through synthesizing active biomolecules such as adipokines and adiponectines and releasing them into the circulation [24]. Adipokines and adiponectines are the two main families of inflammatory cytokines produced and released by adipocytes [7, 8]. After discovering leptin in 1994, hundreds of these peptides have been detected and isolated [25, 26]. These adipocyte-derived biomolecules are closely involved in glycemic control since they may impair or enhance normal signal transduction of insulin in peripheral tissues [26–28]. While adipokines commonly impair insulin sensitivity through inflammation, adiponectines improve it via their anti-inflammatory effects [8, 28]. Although there are some overlapping effects between adipokines and adiponectines, adipokines are classified as insulin-sensitizers (i.e., visfatin, ASP (Acylation-stimulating protein), apelin, adiponectines, and FGF-21 (fibroblast growth factor-21)) and adiponectines as insulin-antagonizers (i.e., TNF-*α* (fibroblast growth factor-21) (produced mainly by macrophages and lymphocytes, but also by other cell types, including adipocytes), IL-6 (Interleukin 6), IL-2, and resistin) [29, 30].

## 4. Incretins and Adipocytokines

Incretin-based drugs have been shown to affect biological peptides such as adipokines and adiponectines [31, 32]. In the following sections, we will detail what is known of the relationships of these antidiabetic agents with the most important adipokines.

## 5. Leptin

Leptin, or satiety hormone, is a peptide mainly secreted by adipocytes and enterocytes that affect energy balance by control of appetite and feeding [33]. The first discovered adipokine indicated that adipose tissue was not passive storage, but rather an endocrine organ [34]. Leptin acts as an essential signal for the brain to control feeding, and loss of its signal is translated to increased food intake and obesity [33], providing a link between energy intake and expenditure to control glycogenesis, lipogenesis, and fat storage; therefore, preventing lipid accumulation, obesity, and downstream-related complications [34]. This balance is lost in obesity and insulin resistance since cellular sensitivity to circulating leptin is diminished in target receptors (i.e., in the arcuate and ventromedial nuclei, as well as other parts of the hypothalamus and dopaminergic neurons of the ventral tegmental area (VTA)) [34].

There are data suggesting incretin-based therapy has close interactions with leptin expression, secretion, or activities [35, 36], interconnected via vagal afferent neurons (VANs) to control feeding and glucose homeostasis [37]. GLP-1 directly induces centric nuclei involved in leptin secretion and feeding behavior [38]. Anini and Brubaker showed that leptin highly induced GLP-1 secretion in a dose-dependent manner in fetal rat intestinal cells, the mouse L cell line (GLUTag), and the human L cell line (NCI-H716) [39]. They found that mice fed with a high fat diet had hyperleptinemia and leptin resistance that treatment with GLP-1 reversed [39]. Tomasik et al. in 2020 reported that the circulating level of leptin was associated with the GLP-1 serum level [40]. They observed that liraglutide significantly reduced serum leptin levels in prediabetic schizophrenia-spectrum disorder patients [40]. Goldsmith et al. in 2015 provided similar evidence indicating GLP-1 administration reduced serum leptin levels in mice [41].

Frøssing et al. 2018 reported that 26 weeks of liraglutide therapy reduced leptin levels in women with polycystic ovary syndrome [42]. Shi and coworkers 2017 showed that exenatide decreased leptin level in type 2 diabetic patients [43]. Lepsen and colleagues 2015 suggested that GLP-1 and leptin cooperate in the weight maintenance and weight loss effects of GLP-1, probably mediated by a decrease in free circulating leptin in obese individuals [44]. This study emphasizes the role of GLP-1 on leptin secretion and suggests that they are both important in lipid metabolism [44]. In another study, Farr and coworkers found that 17 days of GLP-1 therapy reduced serum leptin levels in patients with type 2 diabetes mellitus (T2DM) [45]. They concluded that GLP-1 is involved in the body's energy balance via metabolic hormones like leptin and ghrelin [45]. Similarly, Li et al. 2017 reported that 6 months of sitagliptin therapy reduced leptin plasma level in obese diabetic patients [46]. Moreover, a recent meta-analysis of randomized controlled trials reported that GLP-1RA has inhibitory effects on leptin levels [47]. Overall, it can be seen that GLP-1 therapy affects leptin levels, and some of its metabolic effects are likely mediated by leptin. However, there is no direct evidence to support this, and further clarification studies are required to elucidate the exact molecular interactions between them.

## 6. Ghrelin

Ghrelin, or hunger hormone, is another adipokine peptide that is produced mainly by endocrine cells of the gastrointestinal tract, especially stomach cells [48]. It was discovered as the endogenous ligand of the GHSR (growth hormone secretagogue receptor), but later investigations showed that it is a potent metabolic hormone involved in the control of energy balance, food intake, body weight, adiposity, glucose metabolism, and feeding behaviours [48]. Deregulated levels of ghrelin are involved in the pathophysiology of obesity, adiposity, hyperinsulinemia, insulin resistance, and DM [49, 50].

There are confirmed physiological interactions between endogenous GLP-1 and ghrelin hormone [51]. While GLP-1 is released following feeding, ghrelin levels are increased before food intake, suggesting that ghrelin induces intestinal L-cells to release GLP-1 to prepare the body for incoming food [51]. Gagnon et al. 2015 showed that in the presence of exendin-4, stimulatory impacts of ghrelin on insulin release were completely inhibited in C57BL/6 mice, indicating that the GLP-1R is required for the gluco-homeostatic effects of ghrelin [52]. Another study by Ronveaux and coworkers 2015 suggested that GLP-1 interacts with ghrelin peptide through vagal afferent neurons to promote metabolic pathways [37]. In addition, Lindqvist et al. 2017 showed that ghrelin had regulatory roles on both expression and secretion of GLP-1 in mice [53]. Thus, it seems that endogenous GLP-1 and ghrelin are integrally involved in modulating their metabolic effects. This is further suggested by the work of Babenko et al. 2019, who found that 24 weeks of GLP-1 therapy reduced serum levels of ghrelin in obese T2DM patients [54]. Recently, Skuratovskaia and coworkers in an in silico study found a positive correlation between GLP-1 and ghrelin in patients after LSG (Laparoscopic sleeve gastrectomy) surgery [55].

## 7. Visfatin

Visfatin is a potent adipokine first isolated in 1994 from human lymphocytes as pre-B cell colony enhancing factor (PBEF) [56]. It is expressed in many organs and tissues such as bone marrow, chondrocytes, hepatic cells, muscle, brain, kidney, spleen, testis, and lung, but preferentially in visceral adipose tissue and macrophages [56]. Visfatin acts as a potent proinflammatory cytokine with immunomodulatory effects that is highly expressed in many inflammatory diseases like rheumatoid arthritis, pneumonia, or irritable bowel syndrome [57–59]. In addition, it has complex molecular interactions with metabolic pathways and body homeostasis [60]. Visfatin indirectly modulates metabolic pathways via cellular mediators such as poly (ADP-ribose) polymerase (PARPs), sirtuins (SIRTs), CD38, and CD157 [61, 62]. Visfatin may increase beta cell proliferation, improve insulin sensitivity, enhance glucose uptake, and induce lipogenesis [63]. Visfatin likely binds to the IRs with a similar affinity as insulin and mimics its activities resulting in a prominent role in glucose metabolism [64, 65]. Visfatin has potent modulatory effects on genes involved in lipid homeostasis, such as fatty acid synthase, lipoxygenase, and lipoprotein lipase [60, 66].

Increasing evidence suggests that visfatin acts as a key mediator of the incretin effects [67–69]. Data indicates that the insulinotropic, lipogenic, and glucose-homeostatic properties of incretins are mediated by visfatin [67–69] and that visfatin has modulatory roles on the pleiotropic effects of diabetes-dependent peptides [70].

The effects of incretin-based therapy on visfatin expression/secretion are controversial. Some evidence indicates that GLP-1 suppresses visfatin release [71, 72]. Bala et al. 2011 reported in 100 healthy participants that both insulin and GLP-1 decreased postprandial visfatin levels [71], suggesting that there is a GLP-1/visfatin axis responsible for the rapid suppression of visfatin release upon oral glucose uptake [71]. In addition, Abdelwahed and coworkers 2018 demonstrated that exendin-4 decreases visfatin expression in brain tissue, and this effect mediates the neuroprotective and cognitive enhancer activities of GLP-1 [72]. A recent study by Jin et al. suggested that liraglutide reduced visfatin levels in high fat diet rats [73], and that liraglutide suppressed inflammatory effects of adipokines as well as visfatin leading to greater insulin sensitivity [73]. Likewise, Li et al. 2017 showed that sitagliptin reduces visfatin levels in obese patients with T2DM [46].

Conversely, there is evidence suggesting that GLP-1 increases visfatin levels and upregulates it [74]. For example, Liu et al. 2013 demonstrated in an in vivo study that GLP-1 administration induces visfatin expression via a PKA (Protein kinase A)-dependent pathway in 3T3-L1 adipocytes [74]. They observed that this effect was suppressed by using a PKA inhibitor of H89 [74]. A clinical study in 2014 showed the liraglutide therapy-induced visfatin protein expression level in T2DM patients [75], and another report in 2016 indicated that higher levels of GLP-1 were associated with increased visfatin in maternal and cord blood of participants [76]. Overall, debate remains about the effects of incretin antidiabetic drugs on visfatin that require clarification.

## 8. Resistin

Resistin or adipose tissue-specific secretory factor is a small cysteine-rich peptide that is under the influence of different proinflammatory stimuli, inducing its expression/release and is mainly expressed and released in humans by macrophages (in rodents, it is expressed by adipocytes), and plays an important endocrine role in inflammatory disorders [77]. It binds to the endotoxin receptor TLR4 (Toll-like receptor 4) and an isoform of decorin (a proteoglycan) known as *Δ*DCN [78, 79]. Resistin release is inhibited by thiazolidinediones [78]. Although several biologic activities such as proinflammatory, proangiogenic, and antiapoptotic properties have been related to resistin, its physiologic importance is still not well understood [80]. However, it is accepted that circulatory levels of resistin are important in energy homeostasis [79, 81]. Its levels commonly increase in obesity and DM [82], and it impairs insulin signaling and induces insulin resistance, while blocking it increases glucose uptake and insulin sensitivity [82]. Resistin is able to induce and promote inflammatory pathways involved in different complications such as hypercholesterolemia, asthma, chronic kidney disease, cirrhosis, atherosclerosis, hepatosteatosis, as well as glucose/lipid intolerance, and insulin resistance [77]. Transgenic animals lacking resistin expression were shown to have reduced levels of postprandial blood glucose due to inhibited hepatic gluconeogenesis [83]. Also, exogenous resistin or its overexpression has been associated with adipose tissue inflammation, increased lipolysis and serum-free fatty acid levels, DAG (Diacylglycerol) accumulation in skeletal muscles, hyperinsulinemia, insulin resistance, and glucose intolerance [84–86].

Evidence suggests that GLP-1 levels in plasma interact with resistin peptide [87] with a positive correlation between resistin and incretin hormones in human blood being reported by Niwa et al. in 2016 [76]. In addition, a recent clinical study showed a positive relation between GLP-1 and resistin in diabetes [87].

Resistin may interact with incretins, although with contradictory results as inhibitory or stimulatory effects have been reported [88]. Kim and coworkers in 2007 reported that GIP increases resistin release from 3T3-L1 adipocytes via a p38 MAPK (p38 mitogen-activated protein kinase) and SAPK/JNK (stress-activated protein kinase/Jun amino-terminal kinase) dependent pathways [68]. They concluded that the addition of resistin to differentiated 3T3-L1 adipocytes mimicked the metabolic impacts of incretins, while resistin was recognized as an insulin antagonizer [68]. Díaz-Soto and colleagues in 2014 reported that a short period of liraglutide increased resistin level in T2DM patients [75]. This evidence suggests that incretin-based drugs may increase resistin levels in short-time administration.

There is evidence supporting incretin-based drugs reducing resistin levels so improving insulin signaling [73]. Jin and coworkers in 2020 demonstrated that liraglutide reversed HFD induced hyper-resistinemia in rats [73]. Quan et al. in 2017 found that exenatide therapy significantly decreased resistin level in obese newly diagnosed diabetic patients [89]. Li et al. in 2015 reported that liraglutide downregulated resistin peptide in patients with T2DM [90]. A meta-analysis in 2015 reported that GLP-1 therapy may reduce the risk of atherosclerosis by lowering the level of resistin and its accompanying inflammation [91]. Similarly, a more recent meta-analysis of randomized controlled trials found that GLP-1RAs decrease resistin levels in diabetes [47]. These data highly suggest that incretin-based drugs exert inhibitory effects on resistin expression/secretion though the inconsistency with apparent short-term administration requires clarification.

## 9. Apelin

Apelin or ligand for G-protein-coupled receptor APJ is a neuroendocrine peptide that is extensively expressed in adipocytes and by a number of other tissues such as kidneys, neurons, vessels, myocardium, gonads, lung, liver, pancreatic islets, gastrointestinal tract, and adrenal glands [92]. It has several active isoforms as apelin-36, apelin-17, and apelin-13, each having different biologic functions such as blood pressure control, angiogenesis, feeding behavior, cardiac contractility, cell proliferation, apoptosis, and stress responses [93]. Apelin is derived from mature adipocytes, and it has been recognized primarily as an adipokine and is closely involved in metabolism and energy homeostasis [92]. Apelin synthesis is directly stimulated and dependent on insulin, which explains why obese individuals have higher levels of apelin [94]. Its exact physiologic function is not understood though many studies have reported that plasma level of apelin is increased in obesity with beneficial antiobesity and antidiabetes effects and therefore may have utility in metabolic disorders [95–97]. It was also suggested that apelin has insulinotropic properties [98] with increased glucose uptake through several pathways such as AMPK (AMP-activated protein kinase) and eNOS (Endothelial nitric-oxide synthase) dependent [99], PI3K/Akt (Phosphatidyl inositol 3-kinase/protein kinase B) dependent Glut-4 (Glucose transporter 4) expression/localization [100], and decreased cAMP (Cyclic adenosine monophosphate) [98, 101, 102].

There is little evidence for the dual interactions between incretin-based therapies and apelin peptide [103]. In comparison, apelin may show stronger hypoglycemic effects than incretins [104]. It was shown that apelin induces GLP-1 release in a dose-dependent manner in both in vitro (STC-1 cells) and in vivo (adult rats) models [105], while incretin therapy may induce apelin secretion [103]. Fan et al. in 2015 showed that vildagliptin increased apelin levels in T2DM patients [103], and they concluded that 12 weeks of vildagliptin therapy normalized glycemic indices and improved insulin sensitivity due to the rise in apelin levels [103]. A more recent study showed that incretin therapy might have some further interactions with apelin [106], but more studies are needed.

## 10. Adiponectin

Adiponectin is an anti-inflammatory adipokine synthesized mainly by adipocytes (but also in other tissues as brain, placenta, and muscles) that regulates a number of metabolic pathways involved in glucose and lipid homeostasis [107]. This endocrine peptide plays important modulatory roles in metabolic complications as obesity, DM, metabolic syndrome, atherosclerosis, and nonalcoholic fatty liver disorder (NAFLD) [108, 109]. It has been shown that adiponectin increases insulin sensitivity probably by control of fatty acid oxidation and preventing gluco/lipotoxicity, Glut-4 localization, and suppression of hepatic gluconeogenesis [110–112]. Adiponectin plasma levels are decreased in obesity and by a sedentary lifestyle and increased after aerobic exercise, caloric restriction, and weight loss [113, 114].

Evidence suggests that incretin-based antidiabetes agents have modulatory effects on adiponectin levels [32, 90, 115]. Most data support that incretins increase adiponectin levels. Hosaka et al. in 2009 showed exendin-4 induced adiponectin expression and accompanied with improved inflammatory status and higher insulin sensitivity in 3T3-L1 adipocytes [32]. Bunck and coworkers in 2010 showed exenatide increased adiponectin levels accompanied with improved insulin sensitivity in patients with T2DM [115]. Similarly, Li et al. in 2015 found that liraglutide upregulated adiponectin in T2DM patients [90]. Likewise, Shi et al. in 2017 were also found that exenatide therapy increased adiponectin levels in patients with T2DM [43]. A clinical trial in 2015 confirmed that vildagliptin increased adiponectin in patients with DM [116]. These studies suggest that incretin-based drugs have positive effects on adiponectin synthesize/release, and these effects may explain in part their insulin-sensitizing impact.

## 11. Conclusion

This review has shown that adipokines and adiponectines are modulated by incretin-based pharmacotherapy (Table 2) and may be in part responsible for their pharmacological effects improving glycemic control and improving the overall metabolic profile. However, the effect on adipokines is not fully understood nor fully investigated with no evidence either way on the incretin effects on FGF-2 and ASP, for instance. However, there is evidence for incretins to induce leptin secretion, potentiate metabolic effects of ghrelin peptide to normalize glucose homeostasis, and there are dual interactions between incretins and apelin peptide. The effect on resistin is unclear, but positive effects on visfatin and adiponectin are reported. Overall, incretin therapy appears to have a positive effect on adipokine and adiponectines; however, more studies need to be done to clarify the molecular pathways, pharmacological and physiological effects.

## Figures and Tables

**Table 1:** Approved forms of incretin-based medications.

	Approved forms	Mechanisms of action	Ref.
GLP-1 RA	Exenatide (exendin-4), albiglutide, liraglutide, lixisenatide, semaglutide, dulaglutide	Agonists of intrinsic incretin receptors	[13, 19]
DPP-4i	Sitagliptin, saxagliptin, vildagliptin, linagliptin	Prevent incretin inactivation by inhibition of DPP-4 enzyme	[21, 23]

**Table 2:** current knowledge on effects of incretin-based antidiabetic drugs on adipokines and adipocyte-derived cytokines.

Adipocyte-derived cytokine	Effects of incretin-based drugs	Ref.
Leptin	Increase active levels of leptin and potentiate its metabolic effects	[38–44]
Ghrelin	Unknown clear interactions may potentiate metabolic impacts of ghrelin	[37, 52–55]
Visfatin	Induce visfatin expression/secretion	[74, 75]
	Reduce active levels of visfatin	[46, 71–73]
Resistin	Decrease resistin activities	[47, 73, 89–91]
	Increase resistin and related impacts	[68, 75]
Apelin	Induce apelin release	[103]
Adiponectin	Increase active levels of adiponectines to enhance insulin sensitivity	[32, 43, 90, 115, 116]

## Data Availability

No primary data is associated with this review article.
